# Satisfaction of Physicians Working in Polish Hospitals—A Cross-Sectional Study

**DOI:** 10.3390/ijerph15122640

**Published:** 2018-11-25

**Authors:** Alicja Domagała, Juan Nicolás Peña-Sánchez, Katarzyna Dubas-Jakóbczyk

**Affiliations:** 1 Department of Health Policy and Management, Institute of Public Health, Faculty of Health Sciences, Jagiellonian University Medical College, 31-531 Krakow, Poland; 2 Department of Community Health and Epidemiology, College of Medicine, University of Saskatchewan, Saskatoon, SK S7N5E5SK, Canada; juan.nicolas.ps@usask.ca; 3 Department of Health Economics and Social Security, Institute of Public Health, Faculty of Health Sciences, Jagiellonian University Medical College, 31-531 Krakow, Poland; katarzyna.dubas@uj.edu.pl

**Keywords:** physicians, hospitals, physician satisfaction, factors, survey

## Abstract

Physician satisfaction is a multidimensional concept associated with numerous factors. The objectives of the study were to evaluate the satisfaction of physicians practicing in hospitals in Poland and to identify factors associated with higher levels of satisfaction. A quantitative, cross-sectional survey of Polish hospitals was conducted between March and June 2018. All doctors working in the hospitals invited to the study were asked to fill in an online survey. Fifteen hospitals were included: seven general, five specialist, and three university ones. The total number of questionnaires analyzed was 1003. The questionnaire included 17 items to measure the level of satisfaction, classified into four dimensions: personal, professional, performance, and inherent. The statistical analyses included: assessment of association between levels of career satisfaction and basic demographic and work-related variables; and multivariable logistic regressions, conducted to determine which variables were associated with higher levels of career satisfaction. The mean level of career satisfaction, on a scale from 1 to 6, was 4.1 (SD = 0.69). Respondents reported high levels of inherent satisfaction: mean = 4.4 (SD = 0.66) and a low personal satisfaction: mean = 3.78 (SD = 0.98). 56.6% of respondents reported being satisfied, but only 8.2% reported a higher level of satisfaction (≥5). The satisfaction of Polish physicians is moderate. Gender, numbers of working hours/week, years of work experience, type of hospital, and stage of professional development were the identified factors associated with higher levels of career satisfaction.

## 1. Introduction

Physician satisfaction is a multidimensional concept that has been associated with environmental factors such as working conditions, task variety, workload, and career prospects [1]. Improved awareness of the relevance of physician satisfaction is needed by physicians and their patients, as well as employers [2]. Numerous researchers have emphasized the importance of recognizing physician satisfaction as a relevant problem for healthcare service delivery. Dissatisfaction of physicians highly affects them as it has been linked with burnout [3], self-reported psychiatric symptoms, poorer perceived mental health [4,5], and intention to leave the practice [6]. There is also evidence that the level of physician satisfaction may influence the quality of healthcare services [5,7,8]. Several studies show a positive relationship between high levels of physician satisfaction and patient satisfaction, as well as an association between low levels of physician satisfaction and poor patient compliance with medical treatments [2,7]. Also, high levels of physician satisfaction have been associated with a lower likelihood of medical errors and suboptimal patient care [1,9]. Considering the importance of physicians’ satisfaction to healthcare quality, it is important to investigate associated factors that contribute to or decrease job satisfaction [10,11,12].

The traditional understanding of physicians’ job satisfaction has been in relation to their current practice and daily work, while physicians’ career satisfaction has been understood in relation to their chosen specialties. Furthermore, there is the conceptual model of physicians’ job and career satisfaction that has been developed based on the framework proposed by the SGIM Career Satisfaction Study Group to explain which factors affect physician satisfaction [13]. The cores of this model are: job, personal, family, and community characteristics; these cores can directly affect physician satisfaction. Within the content motivation theory of Abraham Maslow [14], physicians are highly trained healthcare professionals who require the acknowledgment and satisfaction of higher order needs, such as professional growth, life achievement, career development, autonomy, responsibility, creativity, self-esteem, and self-actualization [15,16]. The satisfaction of low- (e.g., income, job security, access to appropriate resources, etc.) and high-order needs must be considered when assessing motivation and satisfaction of individuals [14]. Indeed, appreciating physicians as integral individuals, career satisfaction can be understood as the satisfaction with different dimensions related to one’s medical career.

The current situation of doctors in Poland is very complex: there is a significant shortage of physicians and Poland has the lowest number of practicing doctors per 1000 population within the European Union countries (2.3/1000 in 2015) [17]. Currently, the shortage of physicians is becoming one of the main reasons for limited access to medical services and lengthening of average waiting time. Also, the age of Polish physicians raises concerns: in 2015, approximately 48% of all practicing physicians and 61% of specialists were over 50 years old. Although regulatory salary increases have been implemented in recent years, compared to the high-income countries, doctors’ wages in Poland are still low [18]. Moreover, the migration rate of Polish doctors to EU countries is significant and the level of this phenomenon is estimated as above 7% [19]. The OECD Report “Health at a Glance 2017” confirms the information described above and shows the very difficult and unfavorable situation of Polish physicians in comparison with those in other OECD countries [17].

Physician satisfaction needs to be measured and monitored to improve working and employment conditions and, consequently, to increase employment stability in the Polish healthcare sector. Until now, there has been no research or available data regarding the satisfaction level of Polish doctors working in hospitals, measured with a multidimensional and integral approach. This is a significant gap in this area, especially when approximately 59% of doctors (of the total number of physicians with the valid right to practice) were employed in Polish hospitals in 2016 [20].

Previous research in Poland only explored the satisfaction of family physicians employed in primary care [21,22] or anesthesiologists working in one of the Polish regions [23]. There are also two research targeting cohorts of medical graduates: the first focused on the job satisfaction of physicians of a pensionable age [24] and the second focused on the factors that predict success in one’s medical career [25]. However, there have been no studies evaluating the career satisfaction of physicians working in Polish hospitals. Consequently, the main objectives of this research were to: (1) evaluate the career satisfaction of physicians practicing in hospitals in Poland using a multidimensional approach; and, (2) identify factors associated with higher levels of career satisfaction of physicians working in Polish hospitals.

## 2. Materials and Methods

### 2.1. Study Design and Sampling

A quantitative, cross-sectional survey of physicians working in Polish hospitals was conducted between 5 March and 15 June 2018. The research methodology was approved by the Bioethical Committee of the Jagiellonian University (approval number: 122.6120.290.2016).

The selection criteria of the hospitals invited to participate in the study were: (1) different geographic areas of influence (to reach national distribution), (2) equal in size and no significant differences in terms of the number of physicians/patient beds in three subgroups: general, specialist, and university hospitals and (3) reachable hospital managers to authorize conducting the research (written permission was obtained, signed by hospital managers of all included hospitals). The hospitals were divided into three groups: general, specialist, and university hospitals. Although there are no formal reference levels for hospitals in Poland, these three groups in practice represent the overall structure of the Polish hospital sector. The general ones are usually small (fewer than 10 wards) hospitals, owned by counties and providing care for local communities. The specialist hospitals are bigger units, usually owned by regional authorities and/or bigger cities and providing a broader scope of services. Finally, university hospitals are owned by medical universities, provide multidisciplinary, highly-specialist services, and are involved in the training of medical staff. 

To ensure a geographical representation of Poland, we invited hospitals from different regions as well as from big, medium, and small cities. The target population were physicians working in Polish hospitals, regardless of the position held or form of employment.

Following the Dillman method, personalized e-mails were sent to physicians, providing the objective and implications of the study [26]. Detailed instructions and a link to complete the survey were included. Then, three follow-up e-mails were sent. All e-mails explained the purpose of the research, its scientific and academic nature, and terms of anonymity and confidentiality. Participation was voluntary. Participants had two weeks in total to answer the online survey (the first email with the request was followed by three reminders). To increase the response rate, the study results were offered back to physicians through their hospital managers. This was a non-monetary incentive for participants as well as managers. Moreover, paper copies of the survey were prepared and sent to physicians who experienced problems completing the online version of the survey. Research has confirmed that multiple follow-ups and reminders including a copy of the survey instrument are associated with a higher response rate than surveys that do not use that approach [27]. Our approach and the actions undertaken are in line with methodologies for improving response rates in surveys of physicians [28] and the best practices in surveying physicians proposed by Flanigan and colleagues [29].

Of the 21 hospitals that were invited to participate, 15 hospitals were included in the study: seven general, five specialist, and three university hospitals. Six hospitals were excluded due to poor engagement of physicians in the study (four hospitals) and lack of support from hospital managers to conduct the survey (two hospitals). The 15 hospitals included in the study involved both public and private institutions, 12 and three, respectively. The response rate in our study was 38% (*n* = 1035). In the data analysis, we included only questionnaires with no missing data. The total study sample was 1003 (*n* = 1003).

### 2.2. Questionnaire

A multidimensional approach to measure career satisfaction of physicians has been developed and used in Canada [15,30]. This approach uses a 16-item questionnaire to measure career satisfaction of physicians based on four corner dimensions (4CornerSat): two traditionally researched dimensions: personal and professional satisfaction; and two innovative dimensions: inherent and performance satisfaction, addressing a higher order of hierarchy needs [15,16,31].

In contrast to the traditional framework of job satisfaction (i.e., assessment of physicians’ satisfaction with the current practice and daily work), career satisfaction incorporates the evaluation of satisfaction of higher-order needs as physicians, such as professional growth, career development, autonomy, responsibility, creativity, self-esteem, and self-actualization [15,16].

In Europe, the 4CornerSat questionnaire was adapted and validated in Spain [16], and in Poland two independent translators and a committee of experts completed the adaptation of the questionnaire into the Polish context, including a pilot with local physicians [31,32].

The adapted instrument included 16 items, plus a global item regarding career satisfaction. Each of the items were scored on a scale from very dissatisfied (1) to very satisfied (6), and had four dimensions of career satisfaction (personal, professional, performance, and inherent), originally, with four items for each [15,31].

*1.*  *Personal dimension* (basic needs) refers to the abilities to keep work responsibilities separate from personal life, to sustain satisfying activities in the community, to control work schedule, and the way that the medical practice is managed [15].
*2.*  *Professional dimension* (basic needs) refers to the physician’s relationship with administrators, nurses, level of remuneration, and the authority to have her/his clinical decisions carried out [15].
*3.*  *Performance dimension* (high-order needs) refers to the ability to access resources to treat patients, success in meeting patients’ needs, capacity to keep up with medical advances, and her/his role in organizing programs for patients [15].
*4.*  *Inherent dimension* (basic needs) refers to the doctor‒patient relationship, the diversity of patients that are seen, interaction with other physicians, and career advancement, which taps into the need for interesting and challenging work as a source of motivation [15].

Given the context of Polish hospitals, the researchers of this study decided to add a new item to the instrument—“your interaction and relationship with your direct supervisor(s),” which would be tested in the professional dimension. The Supplementary Materials present the instrument used in this study to measure career satisfaction of physicians.

Levels of physician’s career satisfaction were computed by summing the item scores and dividing them by 17, yielding to scores from 1.00—very dissatisfied to 6.00—very satisfied. Similarly, scores were calculated for the personal, professional, performance and inherent dimensions.

### 2.3. Statistical Analysis

The internal consistency of “satisfaction” was assessed by the Cronbach’s alpha (α) coefficient for the questionnaire, with and without the new item. Also, the Cronbach’s alpha coefficients were reported for each of the four dimensions. A confirmatory factor analysis (CFA) was used to evaluate the fit of the model to the data (with four dimensions and the new item in the professional dimension). The goodness-of-fit of the model was evaluated considering a: Comparative Fir Index (CFI) > 0.90, Root Mean Square Error of Approximation (RMSEA) < 0.08, and a Tucker‒Lewis index (TLI) > 0.90.

The levels of career satisfaction, as well as that of each of the four dimensions, were presented as mean and standard deviation (SD). The Kolmogorov‒Smirnov test was used to test the assumption of normal distribution. The continuous variables were compared between groups using the test for independent sample or one-way analysis of variance (ANOVA). Also, the repeated measures ANOVA with Fisher’s Least Significant Difference (LSD) post hoc test were used to identify statistically significant differences between four dimensions of career satisfaction. Categorical variables were described by percentages and compared using an χ^2^ test. The Pearson’s correlation coefficient (r) or Spearman’s rank correlation coefficient (R) were calculated to assess the relationship between the satisfaction value and age, work experience and total number of working hours per week.

Physicians were classified as “1 = higher levels of satisfaction” if their scores were ≥5.00; otherwise, they were classified as “0 = lower levels of satisfaction”. This classification was applied to the levels of career satisfaction, as well as to each of the dimensions. Then, multivariable logistic regressions were conducted to determine which variables were associated with higher levels of career satisfaction. The independent variables were type of hospital, gender, working experience, children, marital status, being a specialist, performing additional work shift duties, type of employment form, and total number of working hours per week. A backward design strategy was used for each of the models. Results were presented as odds ratios (ORs) with their corresponding 95% confidence intervals (95% CI), as well as adjusted odd ratio (AORs), which refers to the results of the multivariable logistic regressions.

Statistical analyses were performed using SPSS 23.0 (SPSS Inc., Chicago, IL, USA) and R ver. 3.5.0 software. (R Core Team. 2018. R: A language and environment for statistical computing. R Foundation for Statistical Computing, Vienna, Austria).The CFA analysis was performed in R 3.5.0 using the “lavaan” package. The level of significance was *p* < 0.05.

## 3. Results

Among the 1003 participating physicians, the mean age was 43.4 (SD = 11.76) years old, 52% were men (*n* = 518), 76% were in a relationship (*n* = 761), and 68% had children (*n* = 680). In terms of the respondents’ qualifications and workload, the majority had a specialization (*n* = 679, 68%), performed additional shift-work duties (*n* = 775, 77%) and were employed based on a job agreement (*n* = 662, 66.3%). The mean total number of hours worked per week was 60.3 (SD = 16.94) and 65% (*n* = 654) of them worked in at least one additional unit (see Table 1).

There were statistically significant differences among doctors working in the three hospital groups in terms of age, having a specialization, and number of years of work experience—with the highest values observed in the general hospitals group. The mean age of doctors working in these hospitals was 46.6 (SD = 10.84); 74% of them had children and 82% had a specialization. Also, physicians working in general hospitals had the highest mean number of working hours per week, 63.7 (SD = 16.06), and the majority of them (52%) were employed based on a contract. There was a higher share of doctors employed based on a job agreement in university hospitals (79%) (Table 1).

### 3.1. Career Satisfaction of Physicians

In the sample, the questionnaire 4CorserSAT had a really good internal consistency reliability with (α = 0.906) and without the new item (α = 0.902). By dimension, the internal reliability of each dimension was α = 0.82 for the personal, α = 0.80 for professional, α = 0.83 for performance, and α = 0.81 for the inherent satisfaction. The CFA demonstrated a good model fit of the 17-item questionnaire (see the Supplementary Materials) when testing a model with the four predefined dimensions, i.e., the personal (items #12, 13, 16, and 17), professional (items # 8, 9, 11, 14, and 10), performance (items #4, 5, 6, and 7), and inherent (items # 1, 2, 3, and 15) dimensions. The results of the model fit were CFI = 0.954, RMSEA = 0.075 (90%CI = 0.072–0.078), and TLI = 0.944. The personal, performance, and inherent dimension had four items. The professional one had five items including the new item “your interaction and relationship with your direct supervisor(s).”

**Figure 1:**
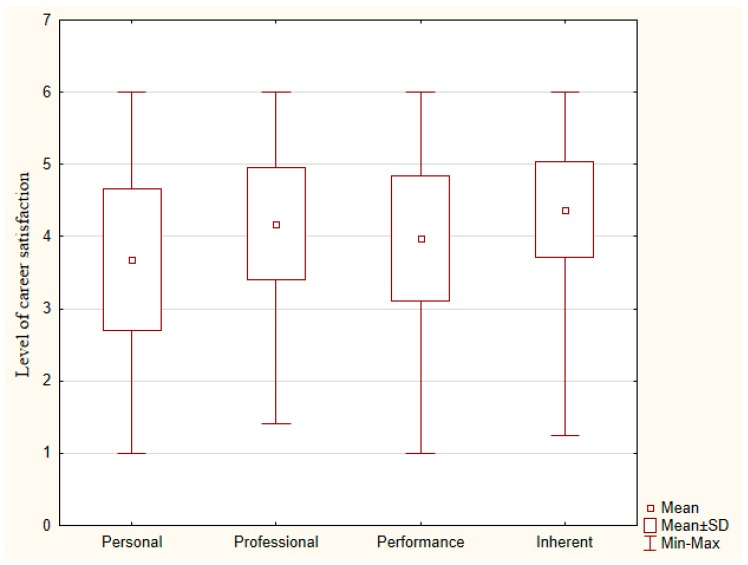
Levels of career satisfaction of physicians working in Polish hospitals by dimensions (*n* = 1003).

On a scale from 1 (very dissatisfied) to 6 (very satisfied), the mean level of career satisfaction of physicians working in Polish hospitals was 4.1 (SD = 0.69). The median career satisfaction was 4.1 (q1 = 3.6, q3 = 4.6). By dimensions, the ANOVA test for repeated measures identified significant differences across the dimension of careers satisfaction (*p* < 0.001), as well as the pairwise comparison. Physicians working in Polish hospitals reported high levels of inherent satisfaction: mean 4.4 (SD = 0.66) and a low personal satisfaction: mean = 3.78 (SD = 0.98) (see Figure 1).

The three items of personal satisfaction had the lowest mean levels, including satisfaction with salary 3.12 (SD = 1.35), life-work balance 3.23 (SD = 1.33), and ability to maintain satisfying non-work-related activities 3.31 (SD = 1.32). In contrast, the highest mean levels of satisfaction were observed among items of the inherent dimension: satisfaction with their interactions with direct supervisor 4.69 (SD = 1.07), interactions with other physicians 4.60 (SD = 0.88), relationships with nurses 4.62 (SD = 0.91), and doctor‒patient relationships 4.35 (SD = 0.83). High levels of satisfaction were also identified in the items of diversity of patients 4.51 (SD = 0.88) and physicians’ success in meeting the needs of patients 4.48 (SD = 0.94). See the Supplementary Materials.

Both age and work experience were positively and significantly associated with overall levels of career satisfaction, as well as with the personal, professional, and performance dimensions of satisfaction. The strongest association was observed between age and professional satisfaction: r = 0.18, *p* < 0.001 (r = 0.19, *p* < 0.001), and overall career satisfaction: r = 0.14, *p* < 0.01 (r = 0.14, *p* < 0.001). In contrast, the number of working hours per week were negatively associated with the satisfaction of physicians, overall career satisfaction (r = −0.12, *p* < 0.001), personal (r = −0.22, *p* < 0.001), and performance (r = −0.11, *p* = 0.001) dimensions. Given that age and years of experience were very strongly correlated (R = 0.98), years of experience was considered in the subsequent analyses to avoid multicollinearity in multivariable model.

By type of hospital, lower mean levels of career satisfaction were observed in university hospitals in comparison to general and specialist ones, *p* = 0.02. Similar differences were observed in the professional (*p* < 0.001) and performance (*p* = 0.03) dimensions, (see Table 2). Furthermore, there was no difference in the levels of career satisfaction between public vs. private hospitals (*p* = 0.49). However, there were differences in the professional and performance dimensions. While physicians in private hospitals had a higher level of professional satisfaction (4.4, SD = 0.61 vs. 4.1, SD = 0.8, *p* < 0.01), their counterparts in public hospitals had higher performance satisfaction (4.0, SD = 0.87 vs. 3.8, SD = 0.82, *p* = 0.04). Details are presented in Table 2.

### 3.2. Factors Associated with Higher Levels of Career Satisfaction

In total, 568 (56.6%) physicians reported a level of career satisfaction at or above 4.00, between somewhat satisfied to very satisfied. Only 82 (8.2%) of physicians reported a career satisfaction level between satisfied and very satisfied, equal or greater than 5.00. By dimensions, 12.4% (personal), 16.5% (professional), 13.9% (performance), and 23.3% (inherent) of physicians reported levels of satisfaction between satisfied and very satisfied (equal or greater than 5.00).

Gender, type of hospital, number of working hours per week and number of years of work experience as well as the stage of professional development (being a specialist) were the identified factors associated with higher levels of career satisfaction among physicians working in Polish hospitals (Table 3). Female physicians were 0.27 (95% CI 0.16–0.48) times less satisfied with their careers that their male counterparts. This association was consistently observed across the four dimension of career satisfaction, identifying marked differences between female and male physicians in the professional and personal dimensions (see Table 3). Similarly, per extra working hour per week, the career satisfaction of physicians decreased by 0.97 (95% CI 0.95–0.98). The impact of numbers of hours per week was consistently observed across the four dimensions of career satisfaction.

On the other hand, physicians working in specialist (AOR = 3.06, 95% CI 1.49–6.30) and university hospitals (AOR = 2.15, 95% CI 1.06–4.36) had a higher odds of reporting higher levels of career satisfaction than those working in general hospitals. This working environment, the type of hospital, had a significant impact on the professional and performance dimensions of physicians satisfaction.

Furthermore, physicians working in Polish hospitals were 1.02 (95% CI 1.01–1.04) times likely to report higher levels of professional satisfaction per each extra year of experience. Specialist physicians had 1.54-fold (95% CI = 1.10–2.18) higher odds of reporting higher levels of inherent satisfaction than physicians in residency training.

## 4. Discussion

This article reports on the first study exploring and analyzing career satisfaction among physicians working in Polish hospitals. We measured career satisfaction in a considerable sample of physicians, over 1000 practicing physicians, working in Polish hospitals.

According to our results, 56.6% of physicians working in Polish hospitals reported being from somewhat satisfied to very satisfied with their careers, although only 8.2% of them report being satisfied to very satisfied with their career.

Our results are in line with other research in this field conducted in European hospitals. According to the results of a systematic review, physician satisfaction in European hospitals is moderate: studies in which data were presented in a dichotomous form show that about 59% of physicians working in European hospitals are satisfied [33].

In terms of satisfaction by each of the four dimensions analyzed, Polish physicians reported higher levels of satisfaction in the inherent dimension, with a mean of 4.4 (SD = 0.66) and the lowest in the personal dimension, with a mean of 3.78 (SD = 0.98). In comparison to the other dimension of career satisfaction of physicians, lower levels of personal satisfaction were similarly reported in Spain [16] and Canada [15], using the same approach to measure physician satisfaction. These results might reflect that, despite differences across healthcare systems, physicians consistently experience difficulties keeping work responsibilities separate from personal life, sustaining satisfying activities in the community, and controlling their work schedule. However, in Poland, these challenges appear to be more critical given that the mean levels of personal satisfaction are lower than those observed in Spain (4.06) [16] and Canada (3.84) [15].

Because of inadequate policies regarding medical staff in Poland, the healthcare system is currently facing serious problems like shortages of medical professionals, excessive workload, emigration of the health workforce and limited access to medical services. The current working and employment conditions of the Polish health workforce are difficult, which results in dissatisfaction and increasing frustration among medical professionals. In May 2018, the National Chamber of Physicians (NCP) conducted an on-line survey concerning doctors’ opinions about their job, the protests of doctors and digitalization of the healthcare system [34,35]. Although the results of this survey should be interpreted with some caution (participants of the survey were members of the NCP working in both in- and outpatient care and the response rate was low), the general outcomes are similar to ours. The results of this survey show that the majority of physicians are satisfied mainly with the effects of their work and patient satisfaction. Almost two-thirds of respondents positively assessed their professional work, more than one in five respondents assessed it as “average,” and every seventh person as “bad.” This confirms the results of our research, in which physicians working in Polish hospitals reported higher levels of satisfaction with their interactions with their direct supervisor, other physicians, nurses, doctor‒patient relationships and success in meeting the needs of patients. The impact of numbers of working hours/week was consistently observed across the four dimensions of satisfaction analyzed.

According to the results of the NCP survey, the greatest physician dissatisfaction was related to the areas of work’s influence on private and family life (45% dissatisfied), level of salary (41% dissatisfied), and working time regulations (41% dissatisfied) [34]. In our research physicians reported the lowest satisfaction level with such aspects as level of salary, life‒work balance, and ability to maintain satisfying non-work-related activities.

Similar results regarding level of physician satisfaction can be found in Hungary [36], Bulgaria [37], Lithuania [38], and Croatia [39]. The healthcare systems in these countries could be classified as the low budget-restricted access type, characterized by a low level of total health expenditure, high private out-of-pocket payments, and limited access to healthcare services [40]. The health systems of these countries are characterized by constant healthcare reform, deficient remuneration, the aging of doctors, health professional emigration, and increasing workloads, which may contribute to physician dissatisfaction. The workforce in these countries has experienced many changes in the health sector, which have generated protests and strikes, which consequently have led to changes in professional attitudes and physician satisfaction. Because of these difficulties, physicians might face numerous barriers to accessing adequate resources for service provision and may experience insufficient payment and incentives, which affect their professional attitude and impact work, job, and career satisfaction.

Age and work experience were positively and significantly associated with overall level of career satisfaction, as well as with all four explored dimensions of satisfaction. The number of working hours per week was negatively associated with the physicians’ satisfaction. Moreover, our results demonstrated statistically significant differences among doctors working in the three hospital groups in terms of age; having a specialization and number of years of work experience, with the highest values observed in the group of general hospitals. These results are a natural consequence of the character of general hospitals, which as relatively small units are less often involved in doctors’ postgraduate education than specialist and, especially, university hospitals. In the case of the latter, there are simply more young doctors being employed. Doctors employed in university hospitals have lower levels of overall as well as professional satisfaction than those working in specialist and general hospitals. However, in terms of the performance dimension of satisfaction, the lowest value was identified in general hospitals. Moreover, physicians working in general hospitals had lower odds of reporting higher levels of career satisfaction than those working in specialist and university hospitals. This might be related to the infrastructure of the general hospitals—a lack of highly specialized equipment and a lower level of financing than in specialist and university hospitals might pose barriers to providing complex care.

The results of our research show that age is positively and significantly associated with overall levels of physician satisfaction, as well as with the four explored dimensions of satisfaction. This confirms the results of previous research conducted by Janus et al. [41] and Rosta et al. [42]. Other studies in which the relationship between physician satisfaction and age was measured did not confirm a statistically significant association [43,44,45]. In the study conducted in Poland by the National Chamber of Physicians, the youngest physicians (up to 37 years old) assessed all job-related aspects much worse than the others. The biggest difference between the average for the youngest doctors and other age groups was recorded in the assessment of remuneration [34].

Another factor identified in our study affecting physician satisfaction was the number of years of work experience (which is naturally connected to age). Previous studies showed mixed results. Some of them indicated that the level of satisfaction decreases with the number of years of experience [46,47]. In turn, Michnov and colleagues reported that long-standing team members reported greater job satisfaction [48]. There are also published studies in which no significant relationship between physician satisfaction and duration of work experience was identified [23,49].

The analysis of the factors associated with higher levels of career satisfaction among physicians working in Polish hospitals identified such factors as: gender, number of working hours per week, number of years of work experience as well as stage of professional development (being a specialist) and type of hospital.

In Europe, previous studies have explored differences in satisfaction between male and female physicians [50]. Some researchers have reported that female physicians tend to be less satisfied than their male colleagues [43,48,49,51,52]. Our study is the first one demonstrating that female doctors are facing similar inequalities in Polish hospitals. We also contribute detailed evidence that marked differences between male and female practitioner can be identified in the satisfaction of lower-order needs of female doctors, specifically in the professional and personal dimensions of career satisfaction. Given that self-actuation cannot be achieved until lower-order needs are satisfied [14], our results highlight the need to reevaluate policies that could be perpetuating gender inequities in Polish hospitals, such as contractual and work environment factors, as well as a call to promote strategies that improve career satisfaction of female physicians.

Polish physicians are dissatisfied with their workload: per extra working hour/week, physician satisfaction decreased by 0.97. Other studies also confirm that physician satisfaction could decline under a heavy workload [51,53,54].

### 4.1. Limitations

This study has some limitations that are necessary to note. The first is the relatively low response rate. Despite our response rate being 38%, a significant amount of international research emphasizes that surveys among physicians have low response rates if compared with the general population [28,29,55]. On the other hand, evidence indicates that medical doctors do not significantly differ among respondents and non-respondents in terms of answers and group characteristics. In this case, larger sample sizes compensate for greater nonresponse [29]. In the case of Polish physicians, one of the reasons for a limited survey response rate could be excessive workload and too much administrative work in the daily practice of medical staff.

According to information from the National Chamber of Physicians, the willingness of doctors to participate in surveys is very low: the percentage of visits to the page with a survey conducted by the NCP among doctors to whom the invitations had been sent was very low (2.7%). Only about 12% of the invited doctors opened the invitation [35]. The highest percentage in all previous studies conducted by the NCP was 17% [35], so our 38% response rate is quite satisfying. However, the biggest challenge for future research projects will be increasing the percentage of doctors participating in the survey.

The next limitation of the survey research is a possible selection bias that could be attributed to the subject of the satisfaction questionnaire, i.e., dissatisfied and frustrated physicians might be especially interested in expressing their opinions and, on the other hand, sensitive doctors might be reluctant to participate in a survey because of fear of identification. Also, the cross-sectional nature of the study precludes causal analysis because it is based only on self-reporting, which could encourage socially desirables answers, and thus does not present a causal relationship between analyzed variables.

Despite these limitations, this is the first study to explore the level of satisfaction of physicians working in Polish hospitals and the associated factors. Our work identified several significant results that could have implications for improving the level of physician satisfaction.

### 4.2. Implications

Understanding the factors affecting physician satisfaction is important not only for medical doctors, but also their patients, healthcare managers, and health policy-makers [41]. Research has demonstrated that physician satisfaction is closely connected with physician wellbeing, quality of healthcare, and patient satisfaction. Moreover, physician satisfaction has a positive impact on patients’ adherence to treatment and actions in managing chronic diseases [2]. On the other hand, dissatisfied physicians tend to have riskier prescribing profiles, fewer adherent patients, less satisfied patients, and possibly decreased healthcare quality [8]. Research has shown that dissatisfied physicians are 2–3 times more likely to leave their profession than satisfied physicians [56]. 

The results of our research could provide useful knowledge for hospital directors to improve the working environment for physicians. Measurement and monitoring of physician satisfaction is very important for policy-makers and healthcare managers in order to develop strategies and improve working conditions in the hospital setting. This is particularly important in the case of Polish doctors, because, as was stated in the introduction, they are in a particularly difficult situation compared to doctors from other European Union countries. Shortages, excessive workload, and poor working and employment conditions may increase doctors’ frustrations, burnout, intentions to migrate, and decision to leave the medical profession. Kravitz concluded that if physician dissatisfaction affects the quality of medical services, physician dissatisfaction is a public health issue [57]. Knowledge and attention regarding levels and factors affecting physician satisfaction should be one of the key issues of health workforce management. Improvement of physician satisfaction will help hospital managers improve their motivation and retention. This study has indicated several significant results that could have implications for improving the level of physician satisfaction in Poland.

## 5. Conclusions

This is the first research investigating the level of physician satisfaction in Polish hospitals and the factors affecting that satisfaction. The satisfaction of doctors working in Polish hospitals is moderate. Physicians reported higher levels of satisfaction in the inherent dimension (satisfaction with their interactions with their direct supervisor, other physicians, nurses, doctor‒patient relationships, and their success in meeting the needs of patients) and the lowest in the personal dimension (level of salary, work‒life balance, ability to maintain satisfying non-work-related activities).

Gender, numbers of working hours per week, and number of years of work experience, as well as the stage of professional development (being a specialist), were the identified factors associated with higher levels of career satisfaction among physicians working in Polish hospitals. Female physicians were less satisfied with their careers than their male counterparts. Similarly, doctors without specialization (under residency training) were less satisfied than specialist physicians. Number of years of work experience was significantly, positively associated with higher levels of career satisfaction. In contrast, the number of working hours/week was negatively associated with physician satisfaction.

## Figures and Tables

**Table 1:** Descriptive statistics.

	Type of Hospital				
	General	Specialist	University		
Variable	*n* = 282	*n* = 325	*n* = 396	ALL (*n* = 1.003)	*p* Value
Age, years, mean (SD)	46.6 (10.84)	41.9 (11.80)	42.34 (11.96)	43.4 (11.76)	<0.001 ^A^
Female, *n* (%)	137 (49%)	159 (49%)	189 (48%)	485 (48%)	0.95 ^B^
Marital status (in a relationship), *n* (%)	213 (76%)	242 (74%)	306 (77%)	761 (76%)	0.67 ^B^
Have children, *n* (%)	208 (74%)	208 (64%)	264 (67%)	680 (68%)	0.03 ^B^
Specialist, n (%)	230 (82%)	190 (58%)	259 (65%)	679 (68%)	<0.001 ^B^
Work experience, years, median (q1–q3)	20.5 (10–28)	12 (5–27)	15 (6–26)	15 (6–27)	<0.001 ^C^
Additional shift-work duties, *n* (%)	228 (81%)	262 (81%)	285 (72%)	775 (77%)	0.005 ^B^
Number of working hours in hospital per week, mean (SD)	46.5 (19.11)	50.7 (13.61)	45.0 (14.5)	47.3 (15.86)	<0.001 ^A^
Total number of working hours per week, mean (SD)	63.7 (16.06)	62.4 (15.44)	56.0 (17.86)	60.3 (16.94)	<0.001 ^A^
Type of employment, *n* (%)					
Job agreement	112 (40.2%)	240 (74.3%)	310 (78.5%)	662 (66.3%)	
Contract	146 (52.3%)	54 (16.7%)	72 (18.2%)	272 (27.3%)	<0.001 ^A^
Mix	21 (7.5%)	29 (8.9%)	13 (3.3%)	63 (6.4%)	

Data are shown as mean (standard deviation = SD), median (q1–q3) or number (percentage). ^A^—*p*-value from ANOVA, ^B^—*p*-value from χ^2^ test, ^C^—*p*-value form Kruskal‒Wallis test.

**Table 2:** Career satisfaction of physicians in Poland by type of hospital and hospital category (*n* = 1.003).

	*n* (%)	Personal	Professional	Performance	Inherent	Overall
**Type of Hospital**						
General	282 (28.1)	3.7 (0.91)	4.3 (0.65) **	3.9 (0.81) *	4.3 (0.6)	4.1 (0.60) *
Specialist	325 (32.4)	3.7 (0.93)	4.3 (0.77) **	4.1 (0.87) *	4.4 (0.66)	4.1 (0.69) *
University	396 (39.5)	3.6 (1.07)	4.0 (0.81) **	4.0 (0.88) *	4.4 (0.71)	4.0 (0.74) *
**Hospital Category**						
Public	834 (83.2)	3.7 (1)	4.1 (0.8) *	4 (0.87) *	4.4 (0.68)	4.1 (0.71)
Private	169 (16.8)	3.7 (0.93)	4.4 (0.61) *	3.8 (0.82) *	4.3 (0.57)	4.1 (0.58)
**Total**	1003 (100)	3.7 (0.98)	4.2 (0.77)	4.0 (0.86)	4.4 (0.66)	4.1 (0.67)

* *p* < 0.05; ** *p* < 0.001.

**Table 3:** Factors associated with higher levels of career satisfaction of physicians in Polish hospitals, overall and by the four dimensions (*n* = 1003).

	Personal Dimension		Professional Dimension		Performance Dimension		Inherent Dimension		Overall Satisfaction	
	OR (95% CI)	AOR 95% CI	OR (95% CI)	AOR 95% CI	OR (95% CI)	AOR 95% CI	OR (95% CI)	AOR 95% CI	OR (95% CI)	AOR 95% CI
Type of Hospital										
General (ref.)	1		1		1		1		1	1
Specialist	1.27 (0.76–2.13)		1.32 (0.88–1.99)	1.71 (1.08–2.71)	1.63 (1.02–2.59)	1.59 (1.01–2.55)	1.14 (0.77–1.69)		2.02 (1.05–3.88)	3.06 (1.49–6.3)
University	1.49 (0.92–2.42)		0.72 (0.47–1.11)	0.78 (0.48–1.26)	1.15 (0.72–1.84)	0.9 (0.55–1.47)	1.41 (0.97–2.03)		1.97 (1.04–3.73)	2.15 (1.06–4.36)
Gender										
Male (ref)	1		1		1		1	1	1	1
Female	0.64 (0.43–0.94)	0.48 (0.32–0.74)	0.46 (0.32–0.65)	0.43 (0.29–0.62)	0.68 (0.48–0.99)	0.53 (0.36–0.78)	0.58 (0.43–0.78)	0.5 (0.36–0.69)	0.39 (0.24–0.64)	0.27 (0.16–0.48)
Work Experience (years)	1.02 (1.01–1.04)		1.03 (1.02–1.05)	1.02 (1.01–1.04)	1.01 (0.99–1.02)		1.02 (1.001–1.03)		1.03 (1.01–1.05)	
Marital Status										
Single (ref.)	1		1		1		1		1	
In relationships	0.95 (0.6–1.49)		1.52 (0.99–2.33)		0.90 (0.59–1.35)		1.27 (0.89–1.80)		1.24 (0.71–2.16)	
Having children										
No (ref)	1		1		1		1		1	
Yes	0.88 (0.59–1.31)		1.53 (1.05–2.25)		0.92 (0.63–1.35)		1.15 (0.83–1.58)		1.32 (0.8–2.2)	
Additional work-shift duties										
No (ref)	1		1		1		1		1	
Yes	0.62 (0.41–0.94)		1.02 (0.69–1.52)		1.03 (0.67–1.58)		1.15 (0.8–1.64)		0.73 (0.44–1.22)	
Stage of professional development										
Before spec. (ref)	1		1		1		1	1	1	
Specialist	1.3 (0.86–1.98)		1.88 (1.26–2.79)		1.04 (0.69–1.55)		1.56 (1.12–2.17)	1.54 (1.1–2.18)	1.91 (1.1–3.32)	
Working hours per week	0.97 (0.96–0.98)	0.96 (0.95–0.98)	0.99 (0.98–1.00)	0.98 (0.97–0.99)	0.98 (0.97–0.99)	0.98 (0.97–0.99)	0.99 (0.98–0.999)	0.98 (0.97–0.99)	0.98 (0.96–0.99)	0.97 (0.95–0.98)
Type of employment form										
Job agreement (ref.)	1	1	1	1	1		1		1	1
Contract	1.52 (1.01–2.28)	1.82 (1.18–2.81)	1.87 (1.31–2.67)	1.64 (1.08–2.49)	1.10 (0.73–1.64)		1.07 (0.77–1.48)		2.01 (1.25–3.22)	2.94 (1.7–5.09)
Mix	0.85 (0.35–2.04)	1.36 (0.55–3.37)	1.03 (0.49–2.17)	1.19 (0.55–2.58)	0.79 (0.35–1.8)		0.86 (0.46–1.64)		0.7 (0.21–2.33)	1.11 (0.32–3.83)

OR: crude odds ratio; AOR = adjusted odd ratios (significant predictors were included in the models using the backward model building strategy); 95% CI = 95% confidence intervals.

## Supplementary Materials

The following are available online at http://www.mdpi.com/1660-4601/15/12/2640/s1, Questionnaire file S1: Please indicate your level of satisfaction with the following aspects of your medical career, Questionnaire file S2: Mean levels of satisfaction, with corresponding standard deviations (SD) and medians for each of the items of the career satisfaction questionnaire (*n* = 1003).
