# Supplementary material for: Satisfaction of Physicians Working in Polish Hospitals—A Cross-Sectional Study
Source: Int J Environ Res Public Health. 2018 Nov 25;15(12):2640. doi: 10.3390/ijerph15122640 (PMC6313796; doi:10.3390/ijerph15122640)
Supplement: Supplementary file 1 [file ijerph-15-02640-s001.pdf]

**SUPPLEMENTARY MATERIAL**

**Supplementary material - file S1. Questionnaire**

***Please indicate your level of satisfaction with the following aspects of your medical career:***

| How satisfied are you with: | | **Very dissatisfied** | **Dissatisfied** | **Somewhat dissatisfied** | **Somewhat satisfied** | **Satisfied** | **Very satisfied** |
|---|---|---|---|---|---|---|---|
| 1 | Your interactions and relationship with other physicians? | [ ] | [ ] | [ ] | [ ] | [ ] | [ ] |
| 2 | The doctor-patient relationships derived from providing patient care? | [ ] | [ ] | [ ] | [ ] | [ ] | [ ] |
| 3 | The diversity of patients you see (age, types of clinical conditions, etc)? | [ ] | [ ] | [ ] | [ ] | [ ] | [ ] |
| 4 | Your success in meeting the needs of your patients | [ ] | [ ] | [ ] | [ ] | [ ] | [ ] |
| 5 | Your ability to access resources needed to treat your patients | [ ] | [ ] | [ ] | [ ] | [ ] | [ ] |
| 6 | Your capacity to keep up with advances in your clinical specialty? | [ ] | [ ] | [ ] | [ ] | [ ] | [ ] |
| 7 | Your role in organizing prophylactic programs for patients? | [ ] | [ ] | [ ] | [ ] | [ ] | [ ] |
| 8 | Your interactions and relationship with nurses | [ ] | [ ] | [ ] | [ ] | [ ] | [ ] |
| 9 | Your interactions and relationship with the hospital administration/management? | [ ] | [ ] | [ ] | [ ] | [ ] | [ ] |
| 10 | Your interactions and relationship with the your direct supervisor | [ ] | [ ] | [ ] | [ ] | [ ] | [ ] |
| 11 | Your authority to get your clinical decisions carried out? | [ ] | [ ] | [ ] | [ ] | [ ] | [ ] |
| 12 | Your ability to control your work schedule? | [ ] | [ ] | [ ] | [ ] | [ ] | [ ] |
| 13 | Your work - personal life balance | [ ] | [ ] | [ ] | [ ] | [ ] | [ ] |
| 14 | Your earnings as a physician? | [ ] | [ ] | [ ] | [ ] | [ ] | [ ] |
| 15 | Your career advancement? | [ ] | [ ] | [ ] | [ ] | [ ] | [ ] |
| 16 | Planning of your career advancements? | [ ] | [ ] | [ ] | [ ] | [ ] | [ ] |
| 17 | Your ability to maintain satisfying non-work related activities (e.g. social, culture)? | [ ] | [ ] | [ ] | [ ] | [ ] | [ ] |
| 18 | Taking into account all factors, you assess your medical career as | [ ] | [ ] | [ ] | [ ] | [ ] | [ ] |

**Supplement file S2**

**Table 1.** Mean levels of satisfaction, with corresponding standard deviations (SD) and medians per each of the items of the Career satisfaction questionnaire (n=1003)

| | **Item of career satisfaction** | **Mean** | **SD** | **Median** |
|---|---|---|---|---|
| 1 | your interactions and relationship with other physicians | 4.60 | 0.88 | 5 |
| 2 | the doctor-patient relationships | 4.35 | 0.83 | 4 |
| 3 | the diversity of patients you see (and their clinical conditions)? | 4.51 | 0.88 | 5 |
| 4 | your success in meeting the needs of your patients | 4.48 | 0.94 | 5 |
| 5 | your ability to access resources needed to treat your patients | 3.87 | 1.14 | 4 |
| 6 | your capacity to keep up with advances in your clinical speciality | 3.98 | 1.10 | 4 |
| 7 | your role in organizing prophylactic programmes for patients | 3.56 | 1.09 | 4 |
| 8 | your interactions and relationship with nurses | 4.62 | 0.91 | 5 |
| 9 | your interactions and relationship with the hospital management | 3.94 | 1.21 | 4 |
| 10 | your interactions and relationship with your direct supervisor | 4.69 | 1.07 | 5 |
| 11 | your authority to get your clinical decisions carried out | 4.52 | 0.99 | 5 |
| 12 | your ability to control your work schedule | 4.18 | 1.13 | 4 |
| 13 | your work - personal life balance | 3.23 | 1.33 | 3 |
| 14 | your salary | 3.12 | 1.35 | 3 |
| 15 | your career advancement | 4.04 | 1.09 | 4 |
| 16 | planning of your career advancements | 3.99 | 1.09 | 4 |
| 17 | your ability to maintain satisfying non-work related activities (e.g. social, culture) | 3.31 | 1.32 | 3 |
